# Protective Effects of Hyperoside on Type 2 Diabetes Through the Regulation of Pancreatic β‐cell Function

**DOI:** 10.1002/iid3.70480

**Published:** 2026-07-07

**Authors:** Yuanyuan Jia, Yincui Ai, Fei Lin

**Affiliations:** ^1^ Department of Endocrinology The Third Affiliated Hospital of Qiqihar Medical University Qiqihar China; ^2^ Department of Nursing Gannan County People's Hospital Qiqihar China

**Keywords:** hyperoside, oxidative stress, pancreatic β‐cells, type 2 diabetes

## Abstract

**Background:**

Type 2 diabetes mellitus (T2D) is a prevalent chronic disease characterized by pancreatic β‐cell loss induced by hyperglycemia. Effective treatments to repair β‐cell damage remain unclear. Hyperoside, a flavonoid glycoside with diverse pharmacological effects, has rarely been studied in H_2_O_2_‐induced β‐cell injury, and its mechanisms are unknown.

**Objective:**

To explore the protective effects of hyperoside on H_2_O_2_‐induced pancreatic β‐cell damage and its underlying mechanisms.

**Methods:**

RIN‐m5F cells were pretreated with 0, 25, 50, 100 μM hyperoside or 5 μM ML385 (inhibitor of the *Nrf2/HO‐1* pathway) for 48 h, followed by 100 μmol/ml H_2_O_2_ for another 30 min. MTT assay and flow cytometry were used to assess cell viability and apoptotic cells. The activities of ROS, CAT, SOD, GSH‐PX, and MDA were measured using the corresponding assay kits. The levels of apoptosis‐related proteins, including cleaved‐caspase3 and caspase‐3, were determined by western blotting. A glucose‐stimulated insulin secretion (GSIS) assay was used to assess insulin secretion. Reverse‐transcription quantitative PCR (RT‐qPCR) and western blotting were used to measure the expression of *Nrf2* and *HO‐1* in the *Nrf2/HO‐1* pathway.

**Results:**

Hyperoside (0, 25, 50, 100 μM) showed no toxic side effects on RIN‐m5F cells. H_2_O_2_ reduced RIN‐m5F cell viability, increased apoptosis, elevated ROS/MDA, decreased antioxidant enzyme activities, suppressed insulin secretion, and downregulated Nrf2/HO‐1. Hyperoside pretreatment reversed these effects, improving viability, reducing apoptosis, restoring antioxidant activity, enhancing insulin secretion, and upregulating Nrf2/HO‐1. ML385 abolished hyperoside's protective effects.

**Conclusion:**

Hyperoside protects pancreatic β‐cells against oxidative stress and apoptosis by activating the Nrf2/HO‐1 pathway. It may serve as a potential therapeutic agent for preventing β‐cell damage in T2D.

## 1 Introduction

Type 2 diabetes, the predominant type of diabetes mellitus, is characterized by insulin resistance and β‐cell dysfunction. More than 90% of diabetes cases are type 2 resulting from defective insulin action or insulin resistance [1]. T2D usually develops due to insulin resistance in peripheral tissues rather than in pancreatic β‐cells, with increasing incidence and mortality rates, affecting about 500 million people worldwide [2]. Pancreatic β‐cells, a unique cell population capable of storing and secreting insulin, lead to the dysfunction of glucose uptake regulation between the blood and various cells. Under the stimulation of glucose, high levels of extracellular glucose molecules are transported into β‐cells [3]. Reactive oxygen species (ROS), a byproduct of mitochondrial respiration in β‐cells, are essential for the maintenance of normal physiological functions [4]. An increasing number of studies have revealed that excessive ROS production may cause cellular DNA damage, eventually leading to cell death, apoptosis, tissue damage, and disease. Xie et al. suggested that pyrocatechol significantly prevented oxidative stress‐induced apoptosis to reduce ROS accumulation in cisplatin‐treated cells [5]. Moreover, Li et al. [6] revealed that *miR‐29c* protects against TNF‐α‐induced HT22 cell damage by reducing ROS production and inhibiting neuronal apoptosis. Persistent oxidative stress caused by the continuous production of ROS is a key factor in the pathogenesis of diabetes. For instance, Zhu et al. demonstrated that silencing *Mcl‐1* enhanced mitochondrial functional loss, oxidative stress, and cardiomyocyte apoptosis, thereby resulting in cardiac dysfunction in diabetic mice [7]. However, the underlying mechanisms of pancreatic β‐cell dysfunction in T2D progression are not fully elucidated.

Hyperoside, a flavonoid found mainly in medicinal plants, has various biological effects, including anti‐inflammatory, anti‐oxidant, and anti‐cancer [8]. Emerging evidence has revealed that HPS plays a protective role in multiple diseases. Qiu et al reported that hyperoside inhibited breast cancer through ROS‐related apoptosis and activation of the Bax‐caspase‐3 axis via *NF‐κB* signaling pathway [9]. Wei et al. suggested that hyperoside prevents DOX‐induced cardiotoxicity by suppressing the *NOXs/ROS/NLRP3* pathway [10]. Furthermore, Liu et al. revealed that hyperoside inhibits renal inflammation by regulating macrophage polarization in T2D mice [11]. However, few studies have explored the mechanism of HPS in pancreatic β‐cell damage in diabetes mellitus. Thus, the role of HPS in T2D and its underlying molecular mechanisms require further investigation.

*Nrf2* is a vital transcriptional regulator that regulates oxidative injury. Previous studies have reported that increased expression of genes, including *NQO1*, *HO‐1, CAT, GST*, and *SOD*, is usually indicative of *Nrf2* transcription factor activation [12]. In addition, the Nrf2/HO‐1 pathway is essential for the maintenance of oxidative stress balance and is involved in a variety of diseases. Sun et al. revealed that RIC alleviates oxidative stress and inflammatory responses through the *Nrf2/HO‐1* pathway, which, in turn, improves neurobehavioral function [13]. Ji et al. demonstrated that carnosol protected against PCOS phenotypes by inhibiting oxidative stress and apoptosis in DHT‐treated KGN cells through activation of the *Nrf2/HO‐1* pathway [14]. Moreover, AKBA protects against AP by reducing oxidative stress in macrophages via the *Nrf2/HO‐1* pathway [15]. Therefore, *Nrf2* antioxidant therapy may be a new therapeutic strategy for the treatment of T2D.

Therefore, in this report, we aimed to investigate the protective effects of hyperoside on H_2_O_2_‐induced pancreatic β‐cell damage and its underlying mechanisms, to clarify whether hyperoside has the potential to be a novel therapeutic drug for T2D.

## 2 Materials and Methods

### 2.1 Cell Culture and Treatment

RIN‐m5F β‐cells were obtained from Shanghai Zhongqiao Xinzhou Biotechnology Co. Ltd (ZQ8505) and cultured in RPMI‐1640 medium (Gibco, Carlsbad, CA, USA) containing 10% FBS, 100 U/mL penicillin and 100 μg/mL streptomycin (Gibco) at 37°C with 5% CO2 in a humidified incubator. Then RIN‐m5F were pretreated with 0, 25, 50, 100 μM hyperoside (1335202, Sigma Aldrich) for 48 h, and then exposed to 100 μmol/mL H_2_O_2_ (323381, Sigma Aldrich) for subsequent experiments.

### 2.2 MTT Assay

After treatment for 48 h, RIN‐m5F cells were seeded into 96‐well plates and incubated for 24 h at 37°C. Then, cells were treated with 10 μL MTT solution (C0009S, Beyotime) and incubated for an additional 4 h. After treatment, the solution was discarded and 100 μL DMSO (D8371, Solarbio) was added to dissolve the formazan crystals. Finally, the optical density (OD) at 570 nm was determined using a microplate reader (Tecan Infinite F50) after 15 min of vibration mixing, following the manufacturer's protocol.

### 2.3 ROS Level

The relative levels of ROS were determined using a Reactive Oxygen Species Detection Kit (S0033; Beyotime, China) according to the manufacturer's protocol. In brief, RIN‐m5F cells were seeded into 96‐well plates and incubated with 1 μM DCFH‐DA at 37°C for 12 h. The medium was then removed and the cells were washed with PBS. The fluorescence intensity was measured at Ex488/Em525 nm using a fluorescence spectrometer (TECAN).

### 2.4 Measurement of MDA, GSH‐PX, SOD and CAT Activity

After treatment, RIN‐m5F cells were cultured in 96‐well plates. Subsequently, the CAT, SOD, GSH‐PX and MDA activity of RIN‐m5F cells were checked by Catalase Assay Kit (E‐BC‐K031‐M, Elabscience), Superoxide Dismutase Assay Kit (E‐BC‐K020‐M, Elabscience), Glutathione Peroxidase Assay Kit (E‐BC‐K096‐M, Elabscience) and Lipid Peroxidation (MDA) Assay Kit (E‐BC‐K025‐M, Elabscience) following the manufacturer's instructions.

### 2.5 FCM Assay

RIN‐m5F cells were seeded in 96‐well plates and assessed using an Annexin V‐FITC Apoptosis Detection Kit (C1062M, Beyotime). The cells were treated with Hyperoside or H_2_O_2_, gently mixed, and resuspended in annexin binding buffer containing Annexin V‐FITC and PI for 20 min at room temperature in the dark. Finally, the number of apoptotic cells was determined using a flow cytometer (BD FACSCalibur) and analyzed using FlowJo software.

### 2.6 Western Blot Assay

Total proteins from RIN‐m5F cells were lysed using RIPA lysis buffer (Thermo Fisher Scientific) on ice for 30 min. The lysates were centrifuged and the supernatants were collected. After determining the protein concentration using a BCA Protein Quantification Kit (Beyotime), each protein was mixed with SDS buffer, boiled for 5 min, fractionated by 12% SDS‐PAGE, and transferred onto a PVDF membrane. After that, the membranes were then blocked with 5% skimmed milk, washed with TBST, and cultivated overnight at 4°C with primary antibodies including anti‐cleaved‐caspase3 (25128‐1‐AP, Proteintech, 1:1000) anti‐Caspase3 (19677‐1‐AP, Proteintech, 1:1000), anti‐*Nrf2* (80593‐1‐RR, Proteintech, 1:1000), anti‐*HO‐1* (66743‐1‐Ig, Proteintech, 1:2000) and anti‐GAPDH (60004‐1‐Ig, Proteintech, 1:50000). After washing three times with TBST, the membranes were cultivated with secondary antibodies (7076/7074, CST 1:1000) for 2 h and detected by Novex™ ECL Chemiluminescent Substrate Reagent Kit (P10300, New Cell & Molecular Biotech Co. Ltd). Relative protein expression was assessed using the ImageJ software (Bethesda, MD, USA).

### 2.7 GSIS Assay

The cell supernatant was obtained by centrifugation at 4°C at 2000 g for 10 min. To detect the insulin secretion, Rat INS ELISA Kit (E‐EL‐R2466, Elabscience) was used following the manufacturer's instructions.

### 2.8 qRT‐PCR Analysis

After treatment with various concentrations of hyperoside, total RNA from RIN‐m5F cells was isolated by TRIzol® reagent (YFXM0011P, YI FEI XUE Biotechnology, Nanjing, China) according to the protocol and reverse‐transcribed into cDNA using a HiScript III RT SuperMix for qPCR (R323, Vazyme). ChamQ Universal SYBR qPCR Master Mix (Q711; Vazyme) with a six‐channel fluorescence real‐time quantitative PCR system (qTOWER2.2; Analytik Jena) was used for real‐time qRT‐PCR. After that, the expression level of *Nrf2* and *HO‐1* was analyzed with 2^−ΔΔCt^ method [16] and *GAPDH* was regarded as the internal control. Primer sequences were listed in Table 1.

**Table 1:** Primer sequences for PCR.

Gene	Primer information	Sequence (5′‐3′)		Length
Rat GAPDH	NM_017008.4	Sense	aacgaccccttcattgacct	164 bp
		Antisense	ccccatttgatgttagcggg	
Rat Nrf2	NM_001399173.1	Sense	tgtcagctactcccaggttg	184 bp
		Antisense	atcaggggtggtgaagactg	
Rat HO‐1	NM_012580.2	Sense	ctggtgatggcctccttgta	232 bp
		Antisense	gatgagtacctcccacctcg	

### 2.9 Statistical Analysis

Statistical analyses were conducted using GraphPad Prism software (version 6.0). All experiments were repeated with three independent biological replicates, each containing three technical replicates (*n* = 3). These results of multiple experiments were reported as the mean ± standard deviation (SD). For multiple‐group comparisons, one‐way analysis of variance (ANOVA) followed by Tukey's post‐hoc test was applied. *p < 0.05, **p < 0.01, ***p < 0.001, ^#^ *p* < 0.05, ^##^ *p* < 0.01, and ^###^ *p* < 0.001 indicated as significant difference. ns means no significant difference (*p* > 0.05).

## 3 Results

### 3.1 Hyperoside on the Cytotoxicity of RIN‐m5F Β‐Cells

**Figure 1:**
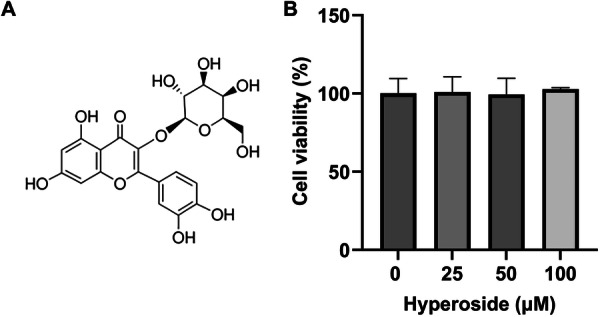
Effects of hyperoside on RIN‐m5F cells. RIN‐m5F cells were stimulated with various concentrations of hyperoside (0, 25, 50, 100 μM). (A) Chemical structure of hyperoside. (B) RIN‐m5F cell viability was assessed by MTT assay. Data are presented as mean ± SD. *n* = 3 biological replicates. Comparisons were analyzed by one‐way ANOVA followed by Tukey's post‐hoc test.

Firstly, RIN‐m5F cells were exposed to various concentrations of hyperoside (0, 25, 50, 100 μM) for 48 h, and the cytotoxic toxic effects of hyperoside on RIN‐m5F cells were measured using the MTT assay. The chemical structure of hyperoside is shown in Figure 1A. Furthermore, the MTT assay demonstrated that hyperoside did not significantly affect RIN‐m5F cell viability (Figure 1B), indicating that hyperoside has no toxic side effects on RIN‐m5F cells.

### 3.2 Hyperoside Significantly Inhibited H_2_O_2_‐Induced Oxidative Stress in Pancreatic Β‐Cells

**Figure 2:**
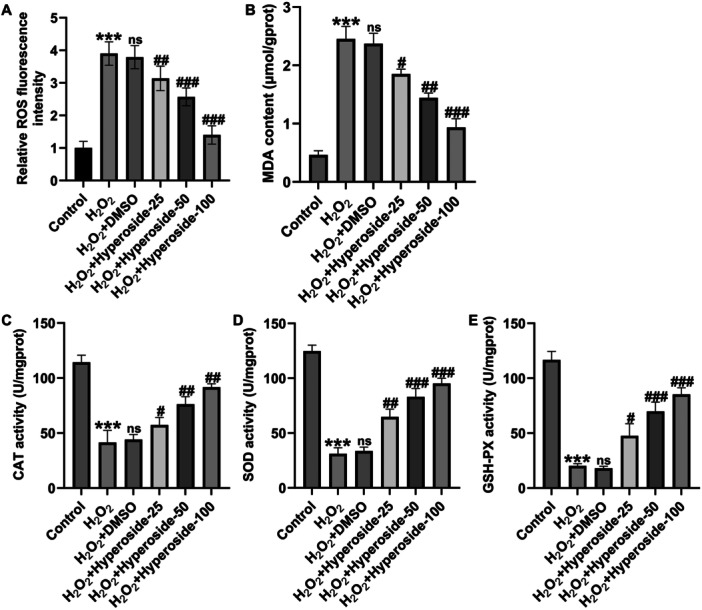
Effects of hyperoside on H_2_O_2_‐induced oxidative stress in RIN‐m5F cells. RIN‐m5F cells were pretreated with 0, 25, 50, or 100 μM hyperoside for 48 h, and then exposed to 100 μmol/ml H_2_O_2_ for another 30 min. The cells were divided into six groups: control, H_2_O_2_, H_2_O_2_ + DMSO, H_2_O_2_+hyperoside‐25, H_2_O_2_+hyperoside‐50, and H_2_O_2_+hyperoside‐100. (A) Intracellular ROS levels (B–D) Determination of CAT, SOD, and GSH‐Px activities. (E) MDA levels in RIN‐m5F cells. Data are presented as mean ± SD. *n* = 3 biological replicates. ***p < 0.001 versus control; ns *p* > 0.05 versus H_2_O_2_; ^#^ *p* < 0.05, ^##^ *p* < 0.01 versus H_2_O_2_ + DMSO (one‐way ANOVA with Tukey's post‐hoc test).

H_2_O_2_‐induced oxidative stress is a vital mechanism that causes cellular damage [17]. Furthermore, we examined whether hyperoside could inhibit H_2_O_2_‐induced pancreatic β‐cell damage, RIN‐m5F cells were pretreated with hyperoside (0, 25, 50, 100 μM) for 48 h, followed by 100 μmol/ml H_2_O_2_ for 30 min. As shown in Figure 2A, H_2_O_2_ elevated ROS levels, whereas ROS production was notably reduced after hyperoside treatment. In addition, H_2_O_2_ exposure caused a significant increase in MDA levels and an obvious decrease in CAT, SOD, and GSH‐Px activities (Figure 2B–E). No significant changes were observed between H_2_O_2_ and H_2_O_2_ + DMSO groups. However, these alterations were ameliorated by hyperoside treatment in a dose‐dependent manner, indicating that hyperoside remarkably suppressed H_2_O_2_‐induced oxidative stress in pancreatic β‐cells and ameliorated cell damage.

### 3.3 Hyperoside Remarkably Suppressed H_2_O_2_‐Induced Apoptosis of Pancreatic Β‐Cells

**Figure 3:**
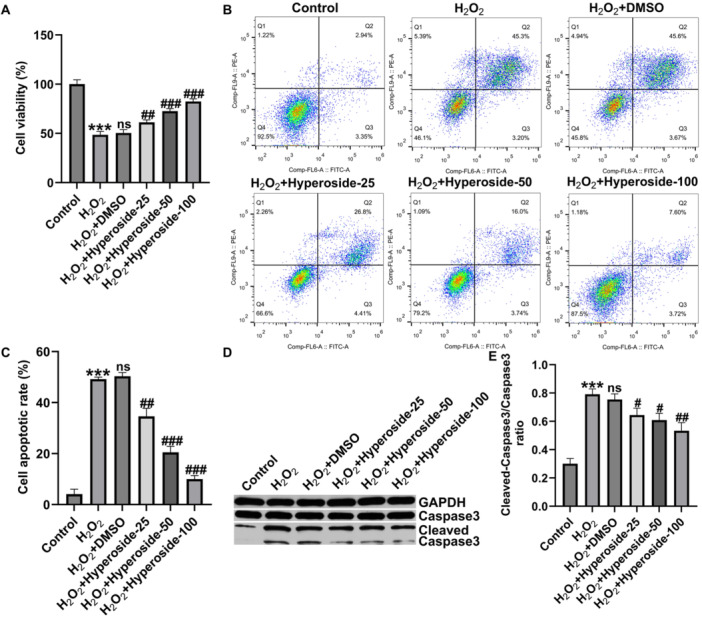
Effects of hyperoside on H_2_O_2_‐induced RIN‐m5F cell viability and apoptosis. The cells were divided into six groups: control, H_2_O_2_, H_2_O_2_ + DMSO, H_2_O_2_+hyperoside‐25, H_2_O_2_+hyperoside‐50, and H_2_O_2_+hyperoside‐100. (A) Cell viability was assessed by MTT assay. (B) Flow cytometry analysis of apoptosis. (C) Quantification of apoptotic cells. (D) Western blotting analysis of cleaved‐Caspase3 and Caspase3 expression. (E) Quantification of cleaved Caspase3/Caspase3 ratio. Data are presented as mean ± SD. *n* = 3 biological replicates. ***p < 0.001 versus control; ns *p* > 0.05 versus H_2_O_2_; ^#^ *p* < 0.05, ^##^ *p* < 0.01, ^###^ *p* < 0.001 versus H_2_O_2_ + DMSO (one‐way ANOVA with Tukey's post‐hoc test).

To investigate the role of hyperoside in pancreatic β‐cell apoptosis in RIN‐m5F cells, MTT and flow cytometry were applied to determine RIN‐m5F cell viability and apoptosis. Compared with the control group, H_2_O_2_ exposure decreased RIN‐m5F cell viability (Figure 3A) and increased the proportion of apoptotic cells (Figure 3B, C). Moreover, we determined the levels of apoptosis‐related proteins, such as cleaved‐caspase3 and caspase3 using western blotting. Our data revealed that cleaved‐caspase‐3 expression was enhanced and the cleaved‐caspase3/caspase‐3 ratio was increased in H_2_O_2_‐induced RIN‐m5F cells (Figure 3D, E), compared with control RIN‐m5F cells. No significant changes were observed between H_2_O_2_ and H_2_O_2_ + DMSO groups. Nevertheless, these effects were partially reversed by hyperoside in a dose‐dependent manner, suggesting that hyperoside plays a protective role against H_2_O_2_‐induced RIN‐m5F cell injury.

### 3.4 Hyperoside Notably Reversed the Effects of H_2_O_2_ on Insulin Secretion in Pancreatic Β‐Cells

**Figure 4:**
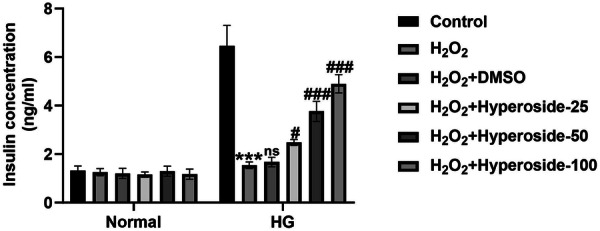
Effects of hyperoside and H_2_O_2_ on high glucose‐stimulated insulin secretion in RIN‐m5F cells. Cells were exposed to 16.7 mM glucose and H_2_O_2_ with or without hyperoside pretreatment for 48 h. The cells were divided into six groups: control, H_2_O_2_, H_2_O_2_ + DMSO, H_2_O_2_+hyperoside‐25, H_2_O_2_+hyperoside‐50, and H_2_O_2_+hyperoside‐100. Insulin secretion capacity was assessed using the GSIS assay. Data are presented as mean ± SD. *n* = 3 biological replicates. ***p < 0.001 versus control; ns *p* > 0.05 versus H_2_O_2_; ^#^ *p* < 0.05, ^###^ *p* < 0.001 versus H_2_O_2_ + DMSO (one‐way ANOVA with Tukey's post‐hoc test).

To illustrate the functional effects of hyperoside on pancreatic β‐cells, the insulin secretion was measured. As shown in Figure 4, H_2_O_2_ treatment decreased high‐glucose (HG)‐stimulated insulin secretion. No significant changes were observed between H_2_O_2_ and H_2_O_2_ + DMSO groups. However, hyperoside blocked H_2_O_2_ stimulated decrease in insulin secretion in a dose‐dependent manner, indicating that hyperoside functionally inhibited the effects of H_2_O_2_ on insulin secretion.

### 3.5 Hyperoside Significantly Reversed the Effects of H_2_O_2_ on the Nrf2/HO‐1 Pathway in Pancreatic Β‐Cells

**Figure 5:**
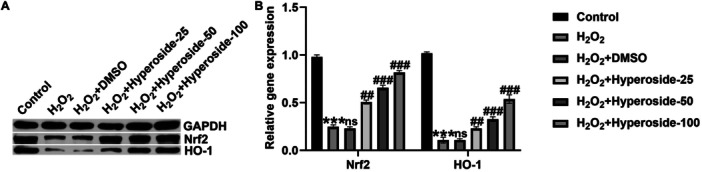
Effects of hyperoside and H_2_O_2_ on Nrf2/HO‐1 pathway in RIN‐m5F cells. RIN‐m5F cells were pretreated with 0, 25, 50, or 100 μM hyperoside for 48 h, and then exposed to 100 μmol/ml H_2_O_2_ for another 30 min. The cells were divided into six groups: control, H_2_O_2_, H_2_O_2_ + DMSO, H_2_O_2_+hyperoside‐25, H_2_O_2_+hyperoside‐50, and H_2_O_2_+hyperoside‐100. (A) Western blotting analysis of Nrf2 and HO‐1 expression. (B) Relative mRNA levels of Nrf2 and HO‐1 were assessed by RT‐qPCR. Data are presented as mean ± SD. *n* = 3 biological replicates. ***p < 0.001 versus control; ns *p* > 0.05 versus H_2_O_2_; ^##^ *p* < 0.01, ^###^ *p* < 0.001 versus H_2_O_2_ + DMSO (one‐way ANOVA with Tukey's post‐hoc test).

A growing number of studies have found that the intracellular antioxidant system is involved in maintaining the cellular redox balance [18]. Next, we investigated the effect of hyperoside on Nrf2/HO‐1 pathway in H_2_O_2_‐induced pancreatic β‐cells. RT‐qPCR and western blotting were performed to detect the expression of antioxidant regulatory proteins including Nrf2 and HO‐1. As expected, H_2_O_2_ reduced the levels of Nrf2 and HO‐1, whereas hyperoside pretreatment enhanced the expression of these proteins in the cells in a dose‐dependent manner (Figure 5A, B). No significant changes were observed between H_2_O_2_ and H_2_O_2_ + DMSO groups. Our data revealed that hyperoside plays a protective role against H_2_O_2_‐induced injury in RIN‐m5F cells by regulating the Nrf2/HO‐1 pathway.

### 3.6 ML385 Reversed the Effects of Hyperoside on Nrf2/HO‐1 Signaling Pathway

**Figure 6:**
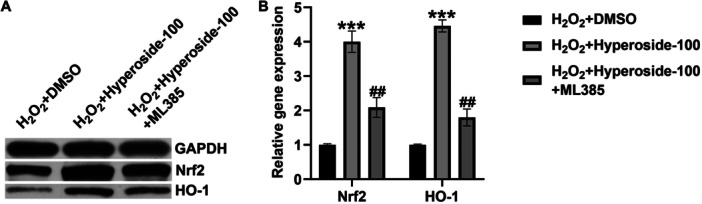
Effects of ML385 on regulation of the Nrf2/HO‐1 signaling pathway. RIN‐m5F cells were pretreated with 100 μM hyperoside or 5 μM ML385 for 48 h, followed by 100 μmol/ml H_2_O_2_ for another 30 min. The cells were divided into three groups: H_2_O_2_ + DMSO, H_2_O_2_+hyperoside‐100, and H_2_O_2_+hyperoside‐100 + ML385. (A) Determination of Nrf2 and HO‐1 expression levels by western blot analysis. (B) RT‐qPCR analysis of Nrf2 and HO‐1 mRNA levels. Data are presented as mean ± SD. *n* = 3 biological replicates. ***p < 0.001 versus H_2_O_2_ + DMSO; ##*p* < 0.01 versus H_2_O_2_+Hyperoside‐100 (one‐way ANOVA with Tukey's post‐hoc test).

In order to explain the roles of Nrf2/HO‐1 pathway in H_2_O_2_‐induced pancreatic β‐cell injury, RIN‐m5F cells were pretreated with 100 μM hyperoside or 5 μM ML385 for 48 h, followed by 100 μmol/ml H_2_O_2_ for another 30 min. Results from Figure 6A and B suggest that H_2_O_2_+Hyperoside‐100 treatment significantly elevated Nrf2 and HO‐1 expression in comparison with the H_2_O_2_ + DMSO group, while this effect was obviously reduced by ML385. Collectively, our findings revealed that hyperoside decreased the levels of Nrf2 and HO‐1 and exerted protective effects.

### 3.7 ML385 Reversed the Effects of Hyperoside on H_2_O_2_‐Induced Oxidative Stress in Pancreatic Β‐Cells

**Figure 7:**
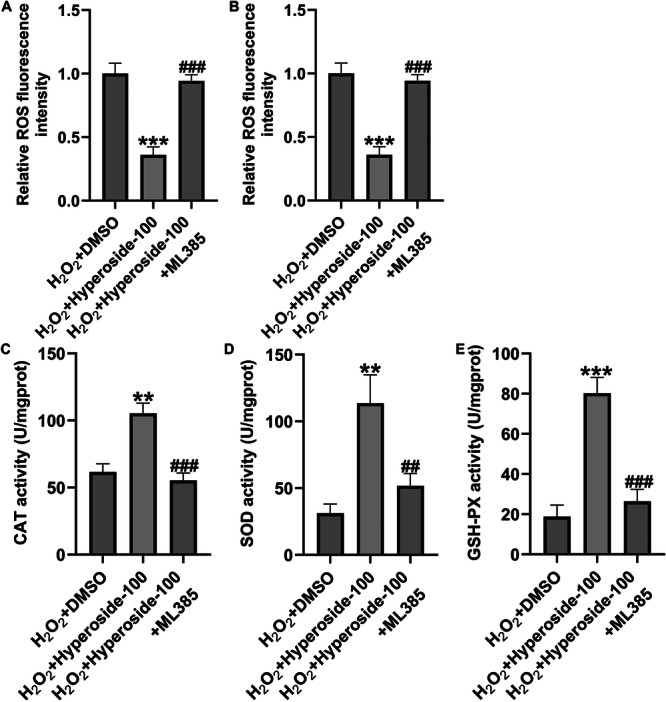
Effects of ML385 and hyperoside on H_2_O_2_‐induced oxidative stress in RIN‐m5F cells. The cells were divided into three groups: H_2_O_2_ + DMSO, H_2_O_2_+hyperoside‐100, and H_2_O_2_+hyperoside‐100 + ML385. (A) Determination of intracellular ROS levels. (B–D) Activities of CAT, SOD, and GSH‐Px were assessed. (E) MDA levels in RIN‐m5F cells. Data are presented as mean ± SD. *n* = 3 biological replicates. **p < 0.01, ***p < 0.001 versus H_2_O_2_ + DMSO; ^##^ *p* < 0.01, ^###^ *p* < 0.001 versus H_2_O_2_+Hyperoside‐100 (one‐way ANOVA with Tukey's post‐hoc test).

In addition, ROS and MDA levels as well as the activities of SOD, CAT, and GSH‐Px were evaluated to explore the effects of ML385 on oxidative stress in H_2_O_2_‐induced RIN‐m5F cells after hyperoside treatment. According to the results in Figure 7, ML385 signally reversed the effects of hyperoside on H_2_O_2_‐induced oxidative stress in pancreatic β‐cells, as evidenced by enhanced ROS production (Figure 7A), increased MDA levels (Figure 7B), and suppressed CAT, SOD, and GSH‐Px activity (Figure 7C–E), as opposed to H_2_O_2_ +Hyperoside‐100 group. Our observations demonstrated that hyperoside suppressed H_2_O_2_‐induced oxidative stress in pancreatic β‐cells by regulating Nrf2/HO‐1 pathway.

### 3.8 ML385 Reversed the Effects of Hyperoside on H_2_O_2_‐Induced Pancreatic β‐cells Viability and Apoptosis

**Figure 8:**
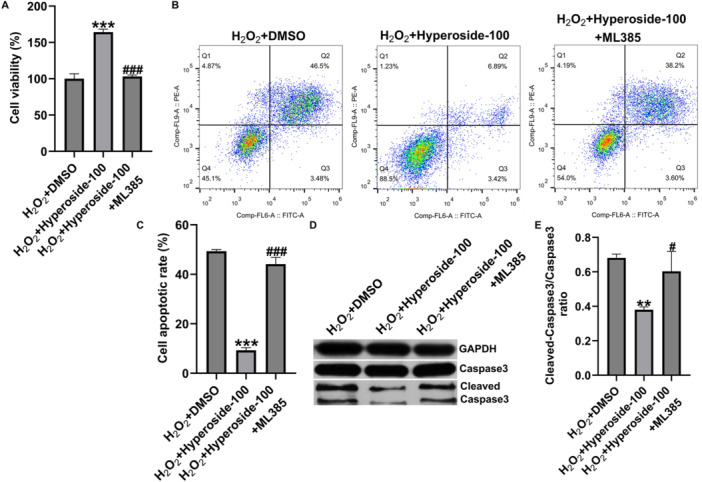
Effects of ML385 and hyperoside on H_2_O_2_‐induced RIN‐m5F cells viability and apoptosis. The cells were divided into three groups: H_2_O_2_ + DMSO, H_2_O_2_+hyperoside‐100, and H_2_O_2_+hyperoside‐100 + ML385. (A) Cells viability was assessed by MTT assay. (B) Flow cytometric analysis of apoptosis. (C) Quantification of apoptotic cells. (D) Cleaved‐Caspase3 and Caspase3 expression were detected by western blotting. (E) Analysis of cleaved Caspase3/Caspase3 ratio. Data are presented as mean ± SD. *n* = 3 biological replicates. **p < 0.01, ***p < 0.001 versus H_2_O_2_ + DMSO; ^#^ *p* < 0.05, ^###^ *p* < 0.001 versus H_2_O_2_+Hyperoside‐100 (one‐way ANOVA with Tukey's post‐hoc test).

To determine whether hyperoside regulates cell viability and apoptosis in H_2_O_2_‐induced RIN‐m5F cells by regulating the Nrf2/HO‐1 pathway, MTT and flow cytometry analyses were performed. We observed that hyperoside treatment promoted RIN‐m5F cell viability (Figure 8A), reduced apoptotic cells (Figure 8B, C), inhibited cleaved‐Caspase3 expression, and depressed the cleaved Caspase3/Caspase3 ratio in H_2_O_2_‐induced RIN‐m5F cells (Figure 8D, E) compared to the H_2_O_2_ + DMSO group. Nevertheless, we found the opposite results in ML385 treated cells, revealing that hyperoside plays a protective role in H_2_O_2_‐induced RIN‐m5F cell injury via the Nrf2/HO‐1 pathway.

### 3.9 ML385 Reversed the Effects of Hyperoside on Insulin Secretion in H_2_O_2_‐Induced Pancreatic Β‐Cells

**Figure 9:**
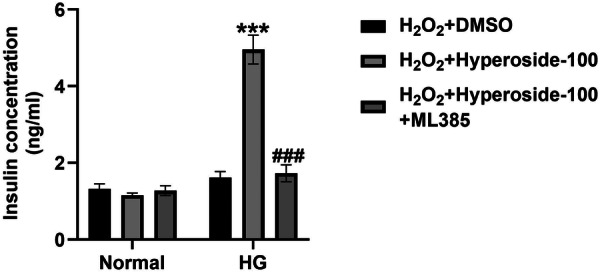
Effects of ML385 and hyperoside on high glucose‐stimulated insulin secretion in H_2_O_2_‐induced RIN‐m5F cells. The cells were divided into three groups: H_2_O_2_ + DMSO, H_2_O_2_+hyperoside‐100, and H_2_O_2_+hyperoside‐100 + ML385. Insulin secretion capacity was assessed using the GSIS assay. Data are presented as mean ± SD. *n* = 3 biological replicates. ***p < 0.001 versus H_2_O_2_ + DMSO; ^###^ *p* < 0.001 versus H_2_O_2_+Hyperoside‐100 (one‐way ANOVA with Tukey's post‐hoc test).

To examining whether ML385 could reverse the insulin secretion in hyperoside treated pancreatic β‐cells, GSIS assay was conducted. Our data revealed that hyperoside dramatically promoted insulin secretion in glucose‐stimulated RIN‐m5F cells compared to the H_2_O_2_ + DMSO group, while this promotion was partly reversed by ML385 treatment (Figure 9). In conclusion, our findings suggest that hyperoside plays a protective role in type 2 diabetes by regulating the Nrf2/HO‐1 pathway.

## 4 Discussion

Diabetes is the third major disease threatening human health after cancer, and cardiovascular and cerebrovascular disease. T2D is a common metabolic disease characterized by systemic metabolic changes, including insulin resistance and hyperglycemia [19]. Insufficient insulin secretion or insulin resistance leads to a series of nutritional and metabolic disorders that can cause hyperglycemia [20]. Hyperglycemia may induce ROS production and oxidative stress in pancreatic β‐cells, resulting in cell damage and inhibited insulin secretion ability [21]. Therefore, oxidative stress plays a vital role in inducing cell damage and promoting the development of T2D. Hyperoside is a well‐known flavonol glycoside that is widely found in medicinal plants. Hyperoside is considered to have a wide range of biological effects, including anti‐fibrotic [22], neuroprotective, and anticancer effects [23, 24]. An increasing number of studies have suggested that hyperoside has protective effects against diabetes. Zhou et al. revealed that hyperoside improved STZ‐induced DN by targeting the miR‐499‐5p/APC axis [25]. Moreover, a report from Chen et al suggested that hyperoside alleviated neuroinflammation, cognitive impairment and oxidative stress by inhibiting TNF‐α/NF‐κB/caspase‐3 signaling in T2D rats [26]. However, the mechanism of action of hyperoside requires further elucidation. This study was designed to explore whether hyperoside has specific effects on the function of pancreatic β‐cells, and to explain its underlying mechanism.

We first investigated the viability of RIN‐m5F cells treated with different concentrations (0, 25, 50, 100 μM) of hyperoside for 48 h to understand the effect of hyperoside on oxidative damage and dysfunction of pancreatic β‐cells induced by H_2_O_2_. Our findings demonstrated that within the concentration range selected in this report, RIN‐m5F cell viability did not change significantly, suggesting that hyperoside had no side effects on RIN‐m5F cells. According to these results and related studies, we selected 25 µM, 50 µM, and 100 µM concentrations of hyperoside for further experiments. Kwon et al. reported that hyperoside reduced intracellular ROS and protected cells from oxidative stress mainly by inducing HO‐1 [27]. This finding was further corroborated by Wang et al., who demonstrated that hyperoside alleviated oxidative stress and IFN production by activating the JNK/Keap1/Nrf2/HO‐1 signaling pathway [28]. STZ has been found to have toxic effects on pancreatic β‐cells, directly damaging their DNA and stimulating the release of H_2_O_2_ [29]. In our study, hyperoside reduced the ROS levels in H_2_O_2_‐induced RIN‐m5F cells in a dose‐dependent manner, indicating that hyperoside may regulate H_2_O_2_‐induced oxidative damage. Lipid peroxidation is the damage caused by ROS to polyunsaturated fatty acids in cellular or organelle membranes. Lipid peroxidation is an oxidative process regulated by enzymatic antioxidants including SOD, CAT, and GSH‐PX. MDA is the main aldehyde product of lipid peroxidation [30]. We observed that H_2_O_2_ disrupted the antioxidant defense mechanisms, leading to significantly decreased intracellular CAT, SOD, and GSH‐Px activities and remarkably enhanced MDA levels. Nevertheless, hyperoside inhibited oxidative stress in H_2_O_2_‐induced pancreatic β‐cells. Consistent with our results, Wang et al. [28] also demonstrated that hyperoside exerts antioxidant effects by activating the Nrf2/HO‐1 pathway. Notably, their study focused on the upstream JNK/Keap1 signaling in an EHV‐8 infection model, while the present work validates the essential role of the downstream Nrf2/HO‐1 cascade in protecting pancreatic β‐cells against H_2_O_2_‐induced oxidative damage, using the Nrf2‐specific inhibitor ML385. Although both studies converge on Nrf2/HO‐1 activation as a core protective mechanism of hyperoside, our findings extend this mechanism to a diabetes‐relevant β‐cell injury model, providing complementary evidence for the antioxidant and cytoprotective actions of hyperoside in distinct pathological contexts.

Researchers have shown that high glucose exposure triggers the apoptotic response of pancreatic β‐cells by promoting ROS formation [31]. Next, we investigated the effect of H_2_O_2_ on pancreatic β‐cells viability and apoptosis. Notably, we found that in H_2_O_2_‐treated RIN‐m5F cells, cell viability was significantly decreased and apoptosis was increased compared to that in the control group. Caspase‐3 is considered the most important terminal protease in apoptosis [32]. Activated caspase‐3 cleaves several substrates to trigger apoptosis. Wei et al. found that hyperoside reduced trastuzumab‐induced myocardial and H9C2 cell apoptosis by reducing the expression of cleaved caspase‐3 and Bax expression [33]. Our study was consistent with this report, showing that the expression of cleaved‐caspase3 and the ratio of cleaved‐caspase3/caspase‐3 in H_2_O_2_‐treated RIN‐m5F cells were enhanced compared with the control group. However, the above findings were reversed after hyperoside pretreatment, indicating that hyperoside has a protective effect on H_2_O_2_‐induced pancreatic β‐cell apoptosis. The excessive formation of H_2_O_2_ can reduce the insulin secretion ability of pancreatic β‐cells [34]. In this study, compared to the control group, the insulin secretion of RIN‐m5F cells was notably decreased under stimulation with 16.7 mM glucose, indicating that the insulin secretion ability was impaired after H_2_O_2_ stimulation. However, hyperoside pretreatment ameliorated H_2_O_2‐_ induced insulin secretion dysfunction in pancreatic β‐cells.

Intracellular antioxidant systems, including the antioxidant regulatory proteins Nrf2 and HO‐1 and the antioxidant protein SOD2, are important for maintaining the intracellular redox balance [35]. To further analyze the specific protective mechanism of hyperoside against H_2_O_2_‐induced oxidative damage, the expression of Nrf2/HO‐1 signaling pathway‐related proteins was analyzed. Compared to the control group, the expression of Nrf2 and HO‐1 in H_2_O_2_ treated cells was significantly decreased, and this inhibition was relieved by hyperoside pretreatment in a dose‐dependent manner. Consistent with the results of this study, Zhu et al. also reported that H_2_O_2_ reduced GC‐2 cell proliferation, reduced the activities of SOD, GSH, and CAT, and enhanced MDA content and the expression of Nrf2 in GC‐2 cells, while hyperoside protected GC‐2 cells from oxidative damage by activating the Nrf2/HO‐1 pathway [36]. Moreover, Xing et al. demonstrated that hyperoside protects against oxidative stress‐induced liver injury by increasing Nrf2 nuclear translocation and promoting HO‐1 expression, suggesting that oxidative stress can be effectively alleviated by activation of the Nrf2/HO‐1 axis [37]. Thus, activation of the Nrf2/HO‐1 pathway may represent a potential target for the treatment of type 2 diabetes.

RIN‐m5F cells were pretreated with 100 μM hyperoside or 5 μM ML385 for 48 h, and then treated with H_2_O_2_ (100 μmol/ml) for 30 min to further explore the regulation of Nrf2/HO‐1 pathway in type 2 diabetes. In this study, hyperoside activated the Nrf2/HO‐1 pathway, which was significantly inhibited by ML385 treatment. Our study further found that ML385 reversed the regulatory effect of hyperoside on pancreatic β‐cells function by increasing ROS production and MDA levels, decreasing CAT, SOD, GSH‐Px activities, decreasing cell viability, increasing apoptotic cells, enhancing cleaved Caspase3 protein expression and cleaved caspase3/caspase3 ratio, as well as inhibiting insulin secretion of cells stimulated by 16.7 mM glucose. These findings suggest that regulation of Nrf2/HO‐1 pathway may contribute to the pancreatic β‐cell function that occurs in type 2 diabetes.

A major limitation of the present study is the lack of *in vivo* experimental validation, combined with the use of an immature *in vitro* β‐cell model. All experiments were performed in the RIN‐m5F cell line, a widely adopted but functionally immature insulinoma model that differs significantly from primary pancreatic β‐cells. RIN‐m5F cells exhibit low endogenous insulin content, blunted glucose‐stimulated insulin secretion (GSIS), and reduced expression of key mature β‐cell transcription factors, including PDX‐1 and particularly MafA, which is barely detectable under basal conditions. Since PDX‐1 and MafA are critical for maintaining β‐cell identity, insulin gene transcription, and physiological secretory function, their deficiency may limit the extrapolation of our findings on the Nrf2/HO‐1 pathway to native β‐cell pathophysiology in type 2 diabetes (T2D). Although our *in vitro* results preliminarily confirmed that hyperoside (HPS) protects β‐cells against H_2_O_2_‐induced oxidative damage via activating the Nrf2/HO‐1 pathway, the *in vitro* monoculture system cannot recapitulate the complex *in vivo* microenvironment of pancreatic β‐cells in T2D. Additionally, this study focused exclusively on the Nrf2/HO‐1 pathway, without exploring other signaling cascades involved in oxidative stress and β‐cell apoptosis, such as the PI3K/Akt, MAPK, and NF‐κB pathways. Oxidative stress‐mediated β‐cell injury is a multifactorial process regulated by multiple interconnected pathways, and hyperoside may exert its protective effects through synergistic modulation of these networks. The lack of investigation into alternative pathways limits the comprehensive understanding of HPS's molecular mechanisms. Furthermore, while hyperoside partially restored H_2_O_2_‐impaired insulin secretion in RIN‐m5F cells, the physiological relevance of these GSIS data is constrained by the cell line's inherently low insulin storage and secretory capacity. Collectively, the in vitro observations require further validation in primary islet β‐cells and *in vivo* T2D animal models (e.g., db/db mice or high‐fat diet combined with streptozotocin‐induced diabetic rats). Future studies will explore the effects of hyperoside on β‐cell function and survival *in vivo*, and confirm whether Nrf2/HO‐1 activation remains the core mechanism in a physiological context. In addition, a known Nrf2 activator was not included as a positive control, and baseline ROS and Nrf2/HO‐1 expression were not examined in the hyperoside‐alone group, which limits the mechanistic interpretation. These aspects will be addressed in future investigations.

To sum up, hyperoside plays a protective role in type 2 diabetes by activating Nrf2/HO‐1 pathway to regulate pancreatic β‐cells function. These findings will help us better understand the role of hyperoside in T2D and may provide new therapeutic strategies for its management. However, the specific roles and potential molecular mechanisms of hyperoside in T2D require further investigation using animal models.

## Author Contributions

**Yuanyuan Jia:** conceptualization, methodology, investigation, writing – original draft, writing – review and editing, data curation. **Yincui Ai:** data curation, supervision, formal analysis, visualization. **Fei Lin:** data curation, writing – review and editing, writing – original draft.

## Ethics Statement

The authors have nothing to report.

## Conflicts of Interest

The authors declare no conflicts of interest.

## Data Availability

The datasets used and/or analyzed during the current study are available from the corresponding author on reasonable request. The data that support the findings of this study are available from the corresponding author upon reasonable request.
